# Composite fiber remodels gut microbiota and shows a trend of alleviating dyspepsia in adolescents with functional dyspepsia: a 16S rRNA pilot study

**DOI:** 10.3389/fmicb.2026.1844725

**Published:** 2026-08-10

**Authors:** Chengrui Yu, Jingsen Lv, Jianli Yang, Yanping Zhang, Ting Yang, Yuan Wei, Yuan Zhang, Jiaying Li

**Affiliations:** 1 Shihezi University School of Public Health, Shihezi, China; 2 Forevergen Biosciences Co, Ltd., Guangzhou, China; 3 Department of Radiology, Changji Branch of the First Affiliated Hospital of Xinjiang Medical University, Changji, Xinjiang, China; 4 Department of Clinical Laboratory, Changji Branch of the First Affiliated Hospital of Xinjiang Medical University, Changji, Xinjiang, China; 5 Department of Pathology, Changji Branch of the First Affiliated Hospital of Xinjiang Medical University, Changji, Xinjiang, China; 6 Department of Oncology, Changji Branch of the First Affiliated Hospital of Xinjiang Medical University, Changji, Xinjiang, China; 7 Department of Colorectal Surgery, Changji Branch of the First Affiliated Hospital of Xinjiang Medical University, Changji, Xinjiang, China; 8 Department of Teaching Research, Changji Branch of the First Affiliated Hospital of Xinjiang Medical University, Changji, Xinjiang, China; 9 School of Food Science and Technology, Shihezi University, Shihezi, Xinjiang, China

**Keywords:** 16S rRNA sequencing, adolescents, composite dietary fiber, functional dyspepsia, gut microbiota

## Abstract

**Background:**

Adolescent functional dyspepsia (FD) is highly prevalent and closely linked to intestinal microbiota imbalance. Dietary fiber exerts prebiotic effects by regulating gut microbiota, but targeted studies investigating whether composite fiber can modulate gut microbiota and alleviate dyspeptic symptoms in adolescent FD patients remain scarce. This pilot trial aimed to explore the gut microbial regulatory characteristics and symptomatic improvement trend of standardized composite fiber supplementation.

**Methods:**

Thirty-two adolescents with FD were enrolled in a 28-day double-blind, placebo-controlled pilot trial (treatment group: *n* = 15, 15 g/d composite fiber; control group: *n* = 17, isoenergetic maltodextrin). Fecal samples were collected post-intervention for 16S rRNA V3-V4 region sequencing. Microbial functional potential was predicted via PICRUSt1, and Fisher’s exact conditional MLE was adopted to compare the binary symptom improvement rates.

**Results:**

Composite fiber elevated all *α*-diversity indices (FDR < 0.05) and separated gut communities (weighted UniFrac PERMANOVA: *F* = 68.3, R^2^ = 0.237, *p* = 0.018), independent of demographics. The intervention was associated with an increased abundance of *Akkermansia*, *Romboutsia*, and [*Eubacterium*]_coprostanoligenes_group, while being associated with a reduction in [*Ruminococcus*]_gnavus_group and *Veillonella*. Notably, cross-sectional FD case–control studies reported contradictory shifts of these genera, and microbial responses to intervention show prominent context-dependent heterogeneity across published research. PICRUSt1-based functional prediction suggested significant upregulation of steroid and flavonoid biosynthesis pathways in the treatment group. The symptom improvement rate was numerically higher in the treatment group (66.7% vs. 47.1%), although this difference was not statistically significant (*p* = 0.3075, OR = 2.19, 95% CI: 0.44–12.15).

**Conclusion:**

Composite fiber intervention reshapes gut microbial diversity, community structure and predicted metabolic functional profiles in adolescents with FD, alongside a non-significant numerical trend toward the alleviation of dyspeptic discomfort. Owing to substantial inter-study heterogeneity and context-dependent microbial responses, the observed genus-level alterations cannot be unequivocally characterized as uniformly beneficial outcomes, nor can direct causal relationships between single microbial taxa and clinical symptom improvement be inferred, as formal mediation analyses were not performed. This pilot study provides preliminary microbiological evidence for a fiber-based non-pharmacological intervention. Large multi-center trials combining multi-omics datasets and formal mediation analysis are required to validate clinical efficacy and untangle complex microbiota-symptom associations.

## Introduction

Functional dyspepsia (FD) represents a widely encountered functional gastrointestinal disturbance in adolescents. It is defined by a constellation of clinical manifestations such as postprandial fullness, belching, anorexia, nausea, and abdominal bloating, and has become a major clinical issue in pediatric gastroenterology. This condition affects adolescents’ daily dietary intake and physical growth, and significantly impairs their quality of life and psychological health (Rosen et al., 2026; Waheed et al., 2026). The etiology of functional dyspepsia (FD) remains intricate and is not yet fully elucidated. Current evidence suggests that gut-brain axis dysregulation and intestinal microecological imbalance are key contributing factors, but their complex interaction hinders a comprehensive elucidation of the underlying mechanisms (Rupp and Stengel, 2022; Chen et al., 2024). Clinically, managing adolescent FD is especially challenging. No identifiable organic lesions are present, and conventional pharmacological therapies have limited efficacy and adverse effects (Yeh and Golianu, 2014). This highlights the urgent need for safer and more effective intervention strategies.

Given these clinical and mechanistic challenges, the gut microbiota has attracted growing interest as a key regulator in functional gastrointestinal disorders (Lee and Lee, 2014; Lee et al., 2017). The gut microbiota is widely recognized as a core “microecological organ” that modulates digestive function, metabolic homeostasis, and immune stability, and is closely linked to gastrointestinal health (Zhang et al., 2025; Colica et al., 2026). A growing body of literature indicates that gut microbiota dysbiosis, defined by diminished microbial diversity, a decline in beneficial taxa, and the proliferation of pro-inflammatory bacteria, is critically linked to both the onset and progression of functional dyspepsia. This dysbiosis can induce visceral hypersensitivity and gastrointestinal dysmotility, the hallmark pathophysiological features of the disorder, thereby exacerbating digestive symptoms (Zhou, 2026; Gomez-Cebrian et al., 2025). Therefore, targeting the gut microbiota to restore microecological balance has become a promising novel strategy for functional dyspepsia, providing a theoretical basis for non-pharmacological interventions.

Among microbiota-targeted interventions, dietary modification stands out for its superior safety and feasibility, with dietary fiber recognized as a particularly promising prebiotic regulator (Montalvan et al., 2025). As an indigestible carbohydrate, dietary fiber acts as the primary fermentable substrate for colonic microbiota, and its intake has been extensively demonstrated to modulate gut microbial structure, enhance beneficial bacterial abundance, and suppress harmful taxa proliferation (Andoh, 2016). Moreover, dietary fiber fermentation generates short-chain fatty acids (SCFAs) such as butyrate, which fortifies intestinal barrier function and mitigates intestinal inflammation, thereby optimizing intestinal microecology and digestive physiology (Liu et al., 2024; Chen et al., 2018). Considering the pivotal contribution of gut microbial imbalance to the pathophysiology of FD, dietary fiber supplementation holds substantial therapeutic potential for relieving digestive discomfort, offering a safe and well-tolerated option tailored to the adolescent population.

Despite the established roles of gut microbiota in digestive health and the therapeutic potential of dietary fiber, critical research gaps persist regarding targeted interventions for adolescent nonspecific digestive discomfort. Most existing studies focus on adult populations or discrete gastrointestinal pathologies such as inflammatory bowel disease, and predominantly utilize generalized dietary assessments instead of controlled trials (Duff et al., 2018; Molendijk et al., 2019). High-quality randomized controlled trials (RCTs) investigating standardized composite dietary fiber interventions in adolescents remain scarce, and few studies have integrated clinical symptom evaluation with high-throughput sequencing-based gut microbiota analysis to clarify the microbial mechanisms underlying fiber-induced symptomatic improvement. This lack of evidence impedes the development of evidence-based dietary guidelines for adolescent digestive disorders.

To fill the above research gap, we carried out a double-blind randomized placebo-controlled external pilot trial. The core exploratory objectives of this preliminary research were: (1) to preliminarily observe the overall changes of gut microbial diversity and community structure in adolescents with FD after standardized composite fiber intervention via 16S rRNA high-throughput sequencing; (2) to screen differential intestinal taxa responding to fiber supplementation; (3) to descriptively analyze the trend of digestive symptom relief and its correlation with microbial remodeling; (4) to accumulate baseline microbial data and optimize the intervention implementation scheme for future adequately powered large-scale formal RCTs.

## Materials and methods

### Study participants

**Figure 1:**
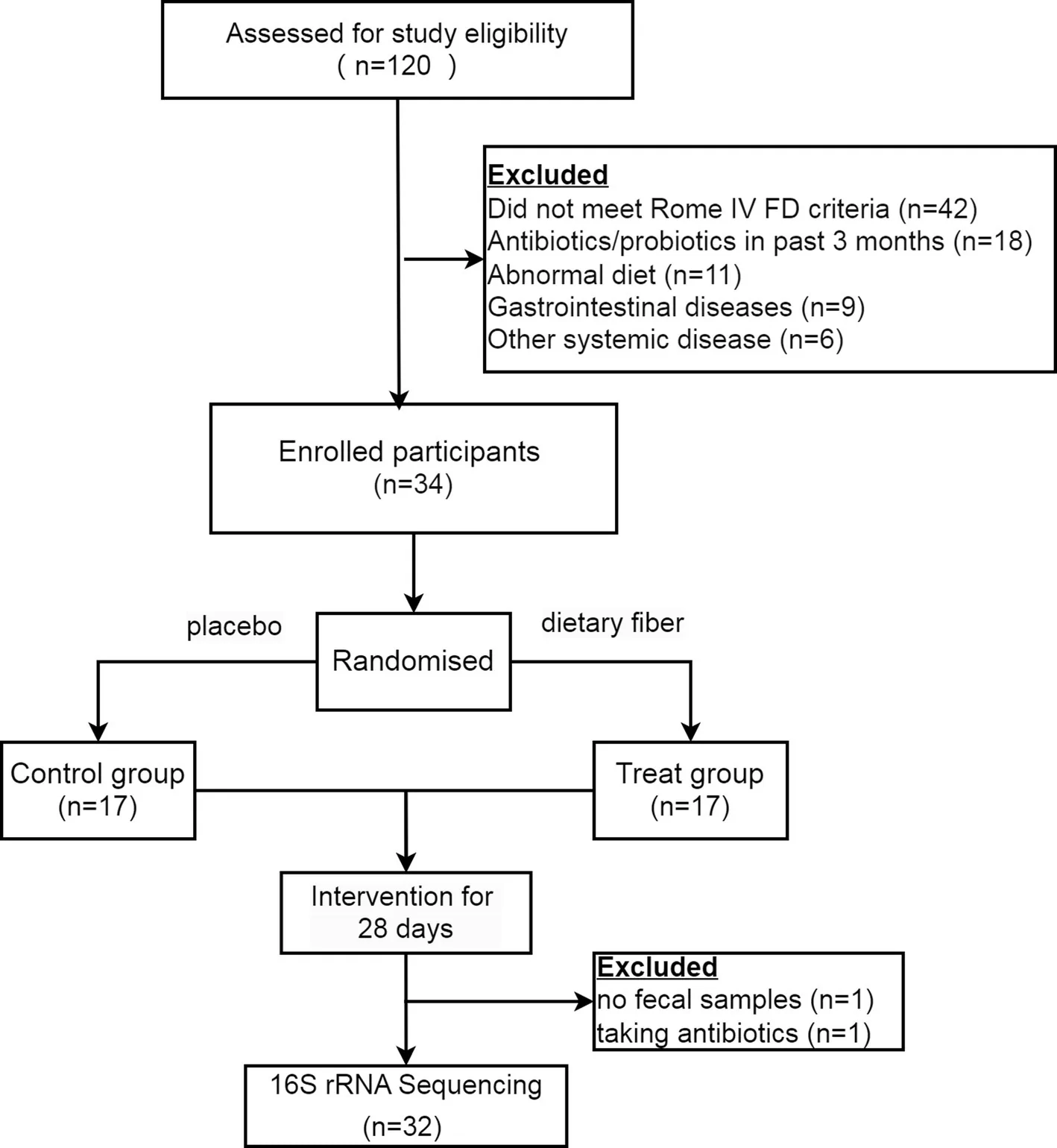
The CONSORT (Consolidated standards of reporting trials) flow diagram depicting patient recruitment scheme for this clinical trial.

A total of 120 adolescents aged between 10 and 19 years presenting with typical dyspeptic symptoms (e.g., postprandial fullness, belching, anorexia/nausea, and abdominal bloating) were initially screened for eligibility from the First Affiliated Hospital of Xinjiang Medical University Changji Branch. All participants were evaluated for FD diagnosis according to the Rome IV diagnostic criteria during screening. Eligible participants fell within the specified age range, had typical dyspeptic symptoms, and voluntarily provided written informed consent to participate. Exclusion criteria included failure to meet the Rome IV criteria for functional dyspepsia, use of antibiotics, probiotics, or prebiotic supplements within the past three months, presence of abnormal dietary habits identified by a preliminary dietary questionnaire, a history of organic gastrointestinal diseases, presence of other concurrent systemic diseases, atypical gastrointestinal symptoms (e.g., severe abdominal pain, hematemesis, melena), and inability to adhere to the intervention and sample collection protocols. After screening, 34 eligible participants were enrolled and randomly assigned (1:1) using a random number table into the control group (placebo, *n* = 17) and the treatment group (dietary fiber, *n* = 17). All participants, investigators, and analysts were blinded to group allocation. The intervention and placebo products were identical in appearance, taste, and packaging. Following the 28-day intervention, two participants were excluded due to failure to provide fecal samples (*n* = 1) and antibiotic use during the trial (*n* = 1); the final analysis included 32 participants (control group *n* = 17, treatment group *n* = 15). A Consolidated Standards of Reporting Trials (CONSORT) flow diagram (Figure 1) illustrates participant flow throughout the trial. Baseline characteristics, including gender, age, body mass index (BMI), education level, food allergy history, and initial severity of digestive symptoms, were compared statistically, with no significant differences observed between groups (all *p* > 0.05; Table 1).

**Table 1:** Baseline characteristics of the Control group and Treat group.

Characteristics	Control group (*n* = 17)	Treat group (*n* = 15)	*p*-value
Female sex	8 (47.1%)	7 (46.7%)	1.0000
Age,yr	14.3 ± 3.3	15.1 ± 2.8	0.4463
BMI, kg/m^2^	20.9 ± 1.3	21.0 ± 1.2	0.8125
Education (above university graduation)	5 (29.4%)	5 (33.3%)	1.0000
Food allergy	0 (0.0%)	1 (6.7%)	0.4688
Postprandial fullness	2.5 ± 0.7	2.4 ± 0.8	0.7998
Epigastric pain	0.2 ± 0.4	0.2 ± 0.4	0.8163
Nausea	1.7 ± 0.7	1.9 ± 0.6	0.4982
Early satiety	2.5 ± 0.7	2.7 ± 0.8	0.4791
Bloating	2.4 ± 0.9	2.5 ± 0.9	0.8637

Prior to participant recruitment, the sample size was justified based on established methodological guidelines for external pilot randomized trials published by Whitehead et al. (2016). As an exploratory pilot study whose primary aim was to characterize gut microbiota alterations rather than definitively test clinical or microbial treatment effects, we followed widely accepted established conventions for two-arm pilot interventional research: the optimal total sample range is 20–40 evaluable participants, with two recommended minimum criteria (≥30 total subjects and at least 10 participants per group) to ensure reliable estimation of microbial variance for future large-scale definitive trials. To accommodate anticipated dropout, fecal sample loss, and protocol violations (approximately 10% exclusion rate), we initially enrolled 34 adolescents. No formal *a priori* power calculation was performed, consistent with standard pilot trial design principles that prioritize feasibility and parameter estimation over formal hypothesis testing.

### Intervention design

The treatment group was supplemented with 15 g/d of Licun® composite dietary fiber produced by Wuxi Licheng Medical Nutrition Co., Ltd. The product consisted of 90 g of dietary fiber per 100 g, composed of polydextrose, inulin, and resistant dextrin. The control group received 15 g/d isoenergetic maltodextrin placebo with identical appearance and energy content. The total duration of the intervention was 28 days.

To minimize dietary confounding, all participants were prescreened using a dietary questionnaire, and those with irregular or extreme dietary habits were excluded before randomization. During the intervention, participants maintained their usual daily diet, and weekly telephone follow-ups were conducted to monitor compliance and stable dietary patterns. After intervention, the efficacy was evaluated by uniformly trained clinicians who scored the severity of four core dyspeptic symptoms (postprandial fullness, belching, anorexia/nausea, abdominal bloating) according to patients’ symptom description. Each symptom was graded on a 4-point scale (0 = none, 1 = mild, 2 = moderate, 3 = severe). The change in total symptom score before and after treatment was used as the primary efficacy endpoint. The symptom improvement rate was calculated as: (Number of cases with symptom improvement/Total number of cases) × 100%.

### Sample collection and DNA extraction

Fecal samples (5 g) were collected from participants in both the treatment and control groups in the morning after an overnight fast at the conclusion of the 28-day intervention period. The collected samples were immediately stored at −80 °C.

Total genomic DNA was extracted using the QIAamp Fast DNA Stool Mini Kit (Qiagen, Germany) following the manufacturer’s protocol. The samples were fully homogenized with phosphate buffer before extraction. Strict aseptic techniques were followed throughout the extraction process, and disposable consumables were used to prevent sample cross-contamination. The integrity of extracted DNA was verified by 1% agarose gel electrophoresis, and the concentration and purity of DNA were measured using NanoDrop 2000 (Thermo Fisher Scientific, USA), with samples considered qualified if the A260/A280 ratio was between 1.8 and 2.0. High-quality DNA samples were stored at −20 °C for later use.

### 16S rRNA sequencing

The V3-V4 region of 16S rRNA gene was amplified with primers 338F (5’-ACTCCTACGGGAGGCAGCAG-3′) and 806R (5’-GGACTACHVGGGTWTCTAAT-3′). The PCR reaction mixture had a total volume of 25 μL, including 12.5 μL of 2 × Taq PCR Master Mix, 1 μL of each primer (10 μM), 1 μL of template DNA (10–50 ng/μL), and 9.5 μL of ddH_2_O. The PCR reaction conditions were as follows: initial denaturation at 95 °C for 3 min, followed by 30 cycles of denaturation at 95 °C for 30 s, annealing at 55 °C for 30 s, and extension at 72 °C for 45 s, with a final extension at 72 °C for 10 min. PCR products were purified using a DNA purification kit (Axygen, USA), quantified using a Qubit 3.0 fluorometer (Thermo Fisher Scientific, USA), and then sequenced on the Illumina Novaseq 6,000 platform (Illumina, USA).

### Bioinformatics analysis

Each sample had a sequencing depth of at least 30,000 reads. Raw reads were filtered using Trimmomatic to remove low - quality reads (average quality score < 20) and adaptor sequences. Chimeras were removed using UCHIME. Operational taxonomic unit (OTU) clustering (97% similarity) was performed using QIIME 1. This 97% OTU QIIME1 pipeline was pre-specified in the trial analytical plan before sequencing. At the trial design stage, QIIME1 OTU workflows were the mainstream analytical framework for clinical gut microbiome interventional studies. Modern DADA2/Deblur ASV pipelines achieve higher strain-level resolution by eliminating fixed clustering thresholds. The 97% OTU strategy may mask subtle intraspecies variation and create minor classification bias for low-abundance taxa. Taxonomic assignment was conducted against the Greengenes database. For *α* diversity analysis, the Observed species, Chao1, Shannon, and PD whole tree indices were calculated and compared between groups using the Wilcoxon rank sum test with false discovery rate (FDR) multiple testing correction. For *β* diversity analysis, three complementary distance metrics were used: Bray–Curtis dissimilarity, weighted UniFrac, and unweighted UniFrac. Principal Coordinate Analysis (PCoA) and Unweighted Pair Group Method with Arithmetic Mean (UPGMA) clustering were performed to visualize sample distribution and grouping based on each distance matrix. Between-group differences in community structure were tested using Permutational Multivariate Analysis of Variance (PERMANOVA) with 999 permutations. To control for potential confounding effects, stratified PERMANOVA was conducted adjusting for age, sex, and BMI across all three distance matrices. Beta dispersion (PERMDISP) was used to evaluate within-group heterogeneity and assess homogeneity of dispersions across groups. Differential microbial taxa were identified using the Kruskal-Wallis H test combined with Linear Discriminant Analysis Effect Size (LEfSe), applying thresholds of a linear discriminant analysis (LDA) score > 2.0 and a Kruskal-Wallis *p*-value < 0.05. To explore functional potential beyond taxonomic composition, we predicted metabolic pathways using PICRUSt1 at the KEGG Level 3 based on 16S rRNA gene sequencing data.

### Statistical analysis

Statistical analyses were performed using SPSS version 26.0, where statistical significance was defined as a p-value < 0.05 or a false discovery rate (FDR) < 0.05 following multiple testing correction. Normality of continuous variables was assessed using the Shapiro–Wilk test; data following a normal distribution were expressed as mean ± standard deviation and compared using the independent samples t-test, while non-normally distributed data were reported as median (interquartile range) and analyzed using the Wilcoxon rank-sum test. Categorical data were presented as *n* (%). Baseline demographic categorical variables were compared using the standard two-sided Fisher’s exact test.

Binary clinical endpoint (symptom improvement) was defined as 1 = symptom improvement, 0 = no symptom improvement. The control group receiving isoenergetic maltodextrin was set as the reference group for odds ratio calculation. Two-sided Fisher’s exact test and conditional maximum likelihood estimation (MLE) were applied to compute crude odds ratios and corresponding 95% exact confidence intervals based on the full 2 × 2 contingency table: 10 improved/5 unimproved in the composite fiber group (*n* = 15), 8 improved/9 unimproved in the placebo control group (*n* = 17).

## Results

### Participant demographic data

Baseline characteristics were well balanced between groups (all *p* > 0.05, Table 1). Specifically, the proportion of females was 47.1% (8/17) in the control group and 46.7% (7/15) in the treatment group. The mean age was 14.3 ± 3.3 years in the control group and 15.1 ± 2.8 years in the treatment group. BMI was 20.9 ± 1.3 kg/m^2^ in the control group and 21.0 ± 1.2 kg/m^2^ in the treatment group. Regarding educational attainment, 29.4% (5/17) of participants in the control group had completed university education or higher, compared to 33.3% (5/15) in the treatment group. Additionally, the prevalence of food allergies was 0% (0/17) in the control group and 6.7% (1/15) in the treatment group.

Regarding the baseline severity of digestive symptoms assessed via a structured participant-reported symptom score (*p* > 0.05), the control group exhibited a postprandial fullness score of 2.5 ± 0.7, epigastric pain score of 0.2 ± 0.4, nausea score of 1.7 ± 0.7, early satiety score of 2.5 ± 0.7, and bloating score of 2.4 ± 0.9; the treatment group had a postprandial fullness score of 2.4 ± 0.8, epigastric pain score of 0.2 ± 0.4, nausea score of 1.9 ± 0.6, early satiety score of 2.7 ± 0.8, and bloating score of 2.5 ± 0.9. The comparability of baseline characteristics between the two groups minimized the influence of confounding factors on the subsequent analysis of intervention effects (Table 1).

### Comparison of therapeutic effects

Following the intervention, improvements in digestive discomfort symptoms (including postprandial fullness, belching, anorexia/nausea, and abdominal bloating) were evaluated. The symptom improvement rates were 47.1% (8/17) in the control group and 66.7% (10/15) in the treatment group. There was no statistically significant difference between the two groups (Fisher’s exact conditional MLE method, *p* = 0.3075), with the treatment group showing a numerical but non-significant advantage (OR = 2.19, 95% CI: 0.44–12.15, Cramer’s *V* = 0.1972).

### Evaluation of sequencing data validity and microbial species coverage

To verify the reliability of sequencing data and ensure the accuracy of downstream microbial analysis, high-throughput 16S rRNA gene sequencing was conducted on 32 samples, consisting of 17 control samples and 15 treatment samples. Following quality control of the raw data, high-quality valid sequences were acquired. The raw paired-end read count per sample varied from 99,408 to 137,387, demonstrating a high retention rate of clean reads post-quality control. The number of clean tags post-assembly ranged from 97,258 to 134,905, with an average sequence length spanning 406–425 nucleotides (nt) and a stable GC content of 51–57%, aligning with the typical characteristics of gut microbiota 16S sequencing. The final valid sequence count ranged from 83,315 to 131,081, with sample efficiency predominantly clustered between 66.35 and 98.11%. Overall, the data exhibited stable quality and high validity, fulfilling the criteria for subsequent microbial diversity and composition analysis (Table 2).

**Table 2:** Quality control and preprocessing statistics of 16S rRNA sequencing data.

Sample ID	Raw reads (PE)	Clean reads (PE)	Raw tags	Clean tags	AvgLen (nt)	GC (%)	No. of seqs	Effective (%)
C001	136,182	136,179	135,397	134,029	415.83	56.86	131,081	97.8
C002	128,357	128,340	127,483	126,181	416.25	54.81	122,458	97.05
C003	120,168	120,140	119,015	117,135	419.94	54.57	114,926	98.11
C004	123,562	123,555	122,656	121,254	418.47	54.92	118,585	97.8
C005	125,911	125,890	124,949	123,470	423.05	53.33	118,724	96.16
C006	124,341	124,336	123,541	122,285	418.91	55.6	117,292	95.92
C007	99,408	99,408	98,594	97,258	422.88	53.53	84,324	86.7
C008	123,431	123,430	122,583	121,166	419.85	52.21	110,770	91.42
C009	129,847	129,846	128,860	127,247	424.07	55.16	120,503	94.7
C010	116,469	116,467	115,523	114,409	418.05	53.1	101,620	88.82
C011	136,230	136,213	135,103	133,645	414.73	54.18	108,029	80.83
C012	134,598	134,593	133,703	132,152	420.9	53.68	114,106	86.34
C013	128,286	128,280	127,515	126,041	420.96	52.87	105,661	83.83
C014	130,359	130,356	129,611	128,307	411.74	54.02	125,440	97.77
C015	128,456	128,456	127,711	126,270	410.54	54.89	123,668	97.94
C016	137,387	137,379	136,338	134,905	412	54.57	117,786	87.31
C017	120,506	120,504	119,742	118,473	412.01	53.2	102,760	86.74
T001	115,802	115,800	114,910	113,745	407.64	53.84	107,640	94.63
T002	128,702	128,693	127,609	126,236	406.32	54.25	117,681	93.22
T003	121,860	121,844	120,865	119,329	409.97	53.54	98,955	82.93
T004	137,263	137,247	136,058	134,423	413.21	52.88	109,286	81.3
T005	122,756	122,752	121,839	120,262	415.81	54.33	113,496	94.37
T006	127,967	127,960	127,029	125,564	419	53.75	83,315	66.35
T007	120,788	120,788	119,949	118,684	418.3	53.13	104,074	87.69
T008	135,354	135,338	134,408	133,096	418.86	53.63	100,587	75.57
T009	134,939	134,929	133,892	132,578	416.41	53	105,044	79.23
T010	122,524	122,520	121,832	104,671	410.61	55.51	101,283	96.76
T011	136,642	136,639	135,740	134,390	409.45	52.8	120,310	89.52
T012	117,011	117,005	116,238	114,962	412.35	55.42	105,324	91.62
T013	121,141	121,137	120,139	118,858	407.36	53.2	105,877	89.08
T014	120,087	120,084	119,398	118,107	412.65	54.24	115,362	97.68
T015	125,799	125,798	124,972	123,748	419.12	51.52	120,968	97.75

**Figure 2:**
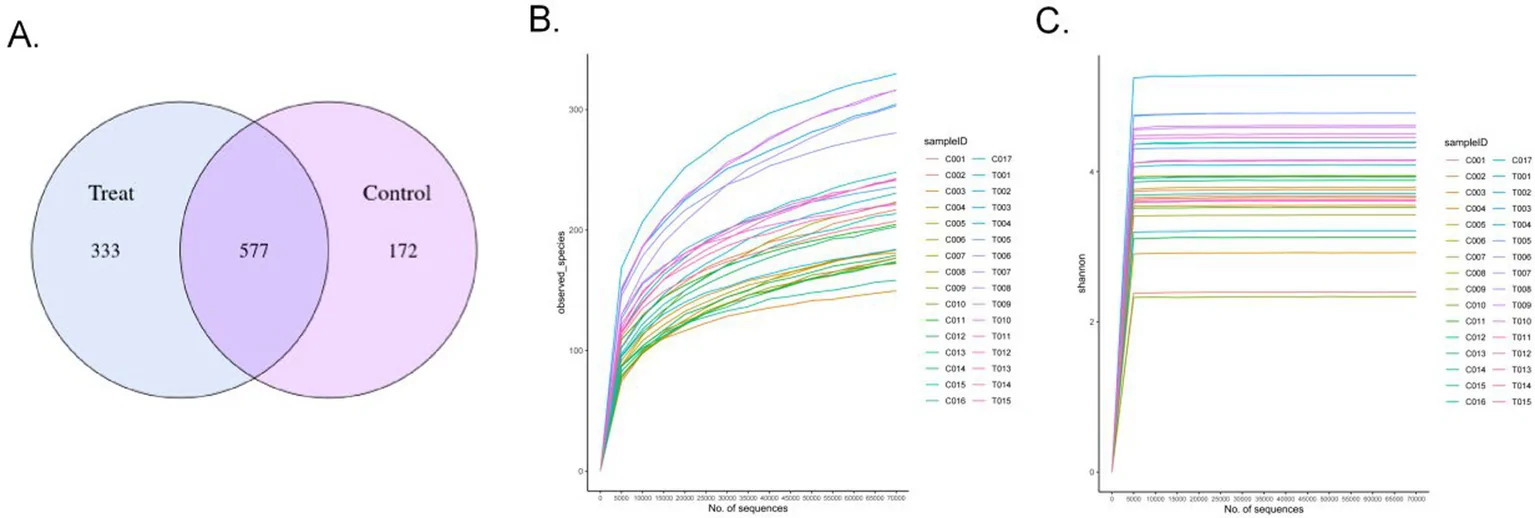
Evaluation of sequencing data validity and microbial species coverage. **(A)** Venn diagram of operational taxonomic units (OTUs) distribution in the gut microbiota of control and treatment groups. **(B)** Rarefaction curve of the Observed_species index. **(C)** Rarefaction curve of the Shannon index.

Based on the validated sequencing data, we further analyzed the distribution of operational taxonomic units (OTUs) and sequencing depth saturation in the two groups. In the control group, 172 unique OTUs were identified, whereas the treatment group yielded 333 unique OTUs, with 577 OTUs shared by both groups (Figure 2A). The rarefaction curves for the Observed_species and Shannon indices reached a plateau (Figures 2B,C), suggesting that the sequencing depth achieved in this study was saturated and adequate to capture the majority of microbial diversity within the samples.

### Comparative analysis of gut microbiota diversity between control and treatment groups

**Figure 3:**
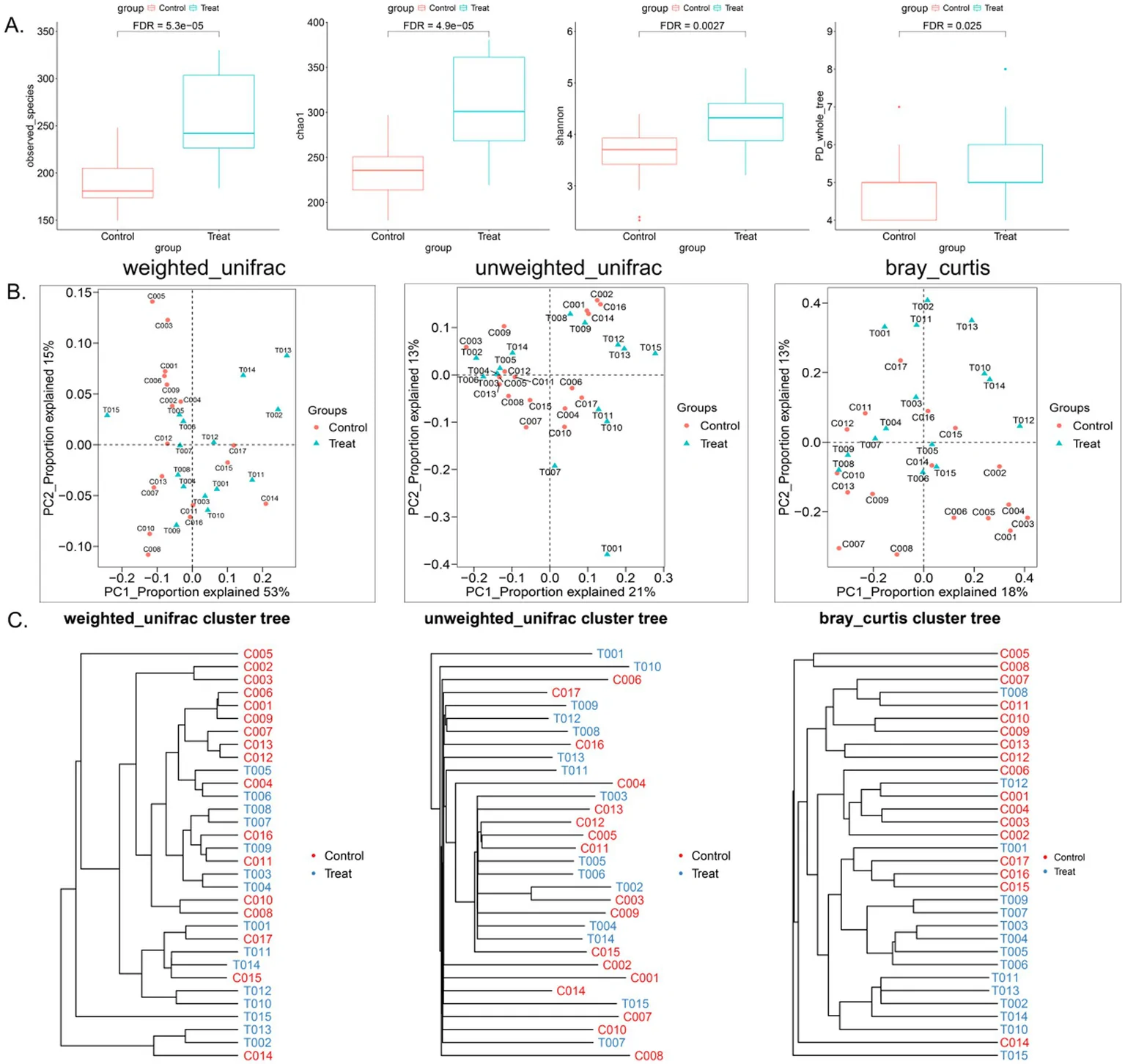
Comparative analysis of gut microbiota diversity between control and treatment groups **(A)** Comparison of *α*-diversity indices of gut microbiota between the two groups. Wilcoxon rank-sum test. **(B)** Principal Coordinate Analysis (PCoA) of gut microbiota *β*-diversity based on the weighted UniFrac distance, unweighted UniFrac distances, and Bray–Curtis dissimilarity **(C)** Unweighted Pair Group Method with Arithmetic Mean (UPGMA) cluster analysis of gut microbiota based on the weighted UniFrac distance, unweighted UniFrac distances, and Bray–Curtis. PERMANOVA and PERMDISP were performed for statistical testing.

Given the sufficient sequencing depth and reliable data quality, we subsequently compared the *α*-diversity and *β*-diversity of intestinal microbiota between the control and treatment groups to explore the effects of dietary fiber intervention. The analysis of gut microbiota diversity revealed that, at the α-diversity level, the treatment group exhibited significantly higher Observed species, Chao1, Shannon, and PD whole tree indices compared to the control group (FDR < 0.05; Figure 3A). These findings suggest that dietary fiber intervention is associated with enhanced species richness, community evenness, and phylogenetic diversity of intestinal microbiota in adolescents diagnosed with FD, indicating a shift toward a more complex community structure.

To further elucidate structural differences in the intestinal microbiota between the two groups, *β*-diversity analysis was conducted using three complementary distance metrics: Bray–Curtis dissimilarity, weighted UniFrac, and unweighted UniFrac. PCoA and UPGMA were generated for each distance matrix to visualize sample separation and clustering (Figures 3B,C). PCoA showed that the cumulative variance explained by the first two axes was 68% for weighted UniFrac, 34% for unweighted UniFrac, and 31% for Bray–Curtis dissimilarity. PERMANOVA based on weighted UniFrac revealed significant differences in community structure (*F* = 68.3, R^2^ = 0.237, *p* = 0.018). PERMANOVA based on Bray–Curtis dissimilarity revealed a trend toward group separation (*F* = 52.3, R^2^ = 0.164, *p* = 0.075), while PERMANOVA based on unweighted UniFrac showed no significant difference (*F* = 72.5, R^2^ = 0.105, *p* = 0.181).

Stratified PERMANOVA was performed to assess the influence of potential confounding factors. For weighted UniFrac, sex (R^2^ = 0.019, *p* = 0.224), age (R^2^ = 0.025, *p* = 0.451), BMI (R^2^ = 0.036, *p* = 0.147), and education (R^2^ = 0.028, *p* = 0.245) explained a negligible and non-significant proportion of the microbiota variation. For Bray–Curtis dissimilarity, sex (R^2^ = 0.021, *p* = 0.253), age (R^2^ = 0.035, *p* = 0.381), BMI (R^2^ = 0.042, *p* = 0.239), and education (R^2^ = 0.039, *p* = 0.302) did not significantly explain variation in community composition. For unweighted UniFrac, sex (R^2^ = 0.005, *p* = 0.351), age (R^2^ = 0.008, *p* = 0.256), BMI (R^2^ = 0.015, *p* = 0.323), and education (R^2^ = 0.011, *p* = 0.187) accounted for no significant variation in community composition. All confounding factors had negligible effects on microbial community profiles.

PERMDISP was applied to assess the homogeneity of multivariate dispersions across groups. All tests yielded non-significant results (weighted UniFrac: *F* = 15.4, *p* = 0.152; Bray–Curtis: *F* = 21.8, *p* = 0.215; unweighted UniFrac: *F* = 25.6, *p* = 0.184), indicating comparable within-group dispersion between the two groups and supporting the reliability of PERMANOVA outcomes.

UPGMA clustering revealed distinct clustering for weighted UniFrac, whereas Bray–Curtis dissimilarity and unweighted UniFrac displayed less defined clustering patterns. This pattern is consistent with the ecological impact of dietary fiber intervention, which mainly modulates the relative abundance of intestinal bacteria rather than causing large-scale species turnover or presence–absence changes.

### Analysis of gut microbiota community structure and differential species between control and treatment groups

**Figure 4:**
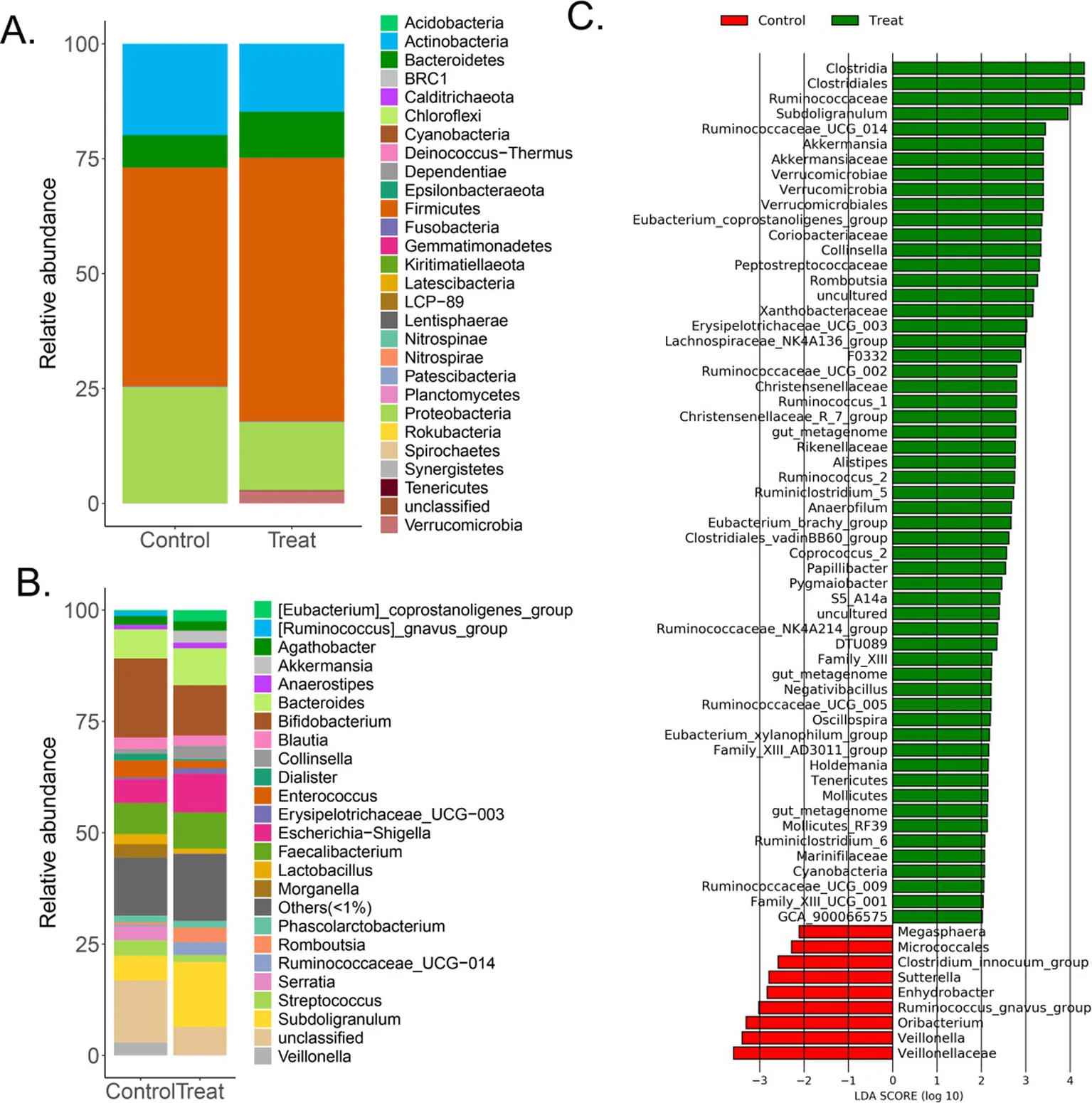
Analysis of gut microbiota community structure and differential species between control and treatment groups. **(A)** Comparative analysis of gut microbiota community makeup at the phylum level. **(B)** Identification of distinct gut microbiota taxa at the genus level. **(C)** Linear Discriminant Analysis Effect Size (LEfse) analysis results. Differential gut microbiota with positive Linear Discriminant Analysis (LDA) scores log10 (green bars) represent taxa enriched in the treatment group, while those with negative scores (red bars) represent taxa enriched in the control group. LDA score > 2.0 and a Kruskal-Wallis *p*-value < 0.05.

**Figure 5:**
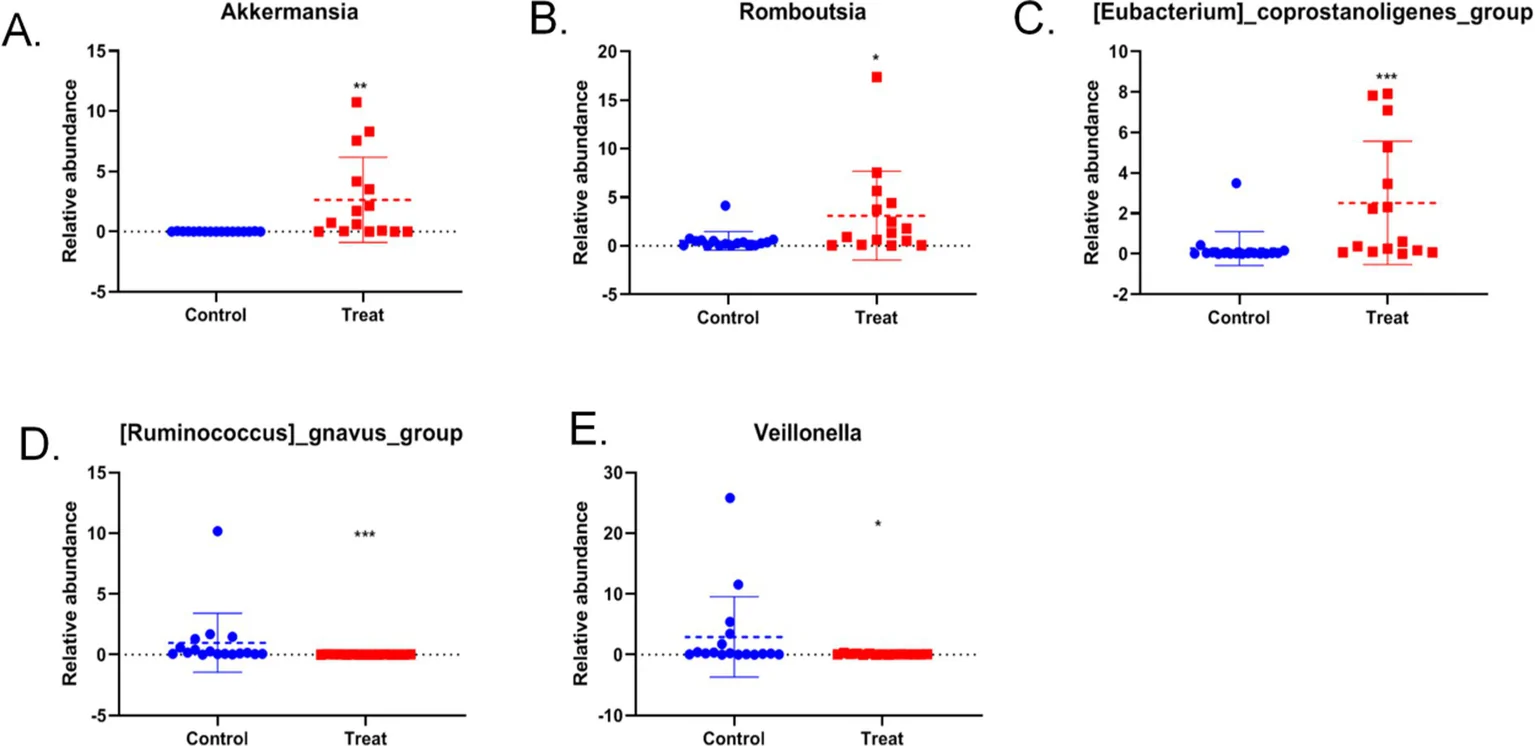
Scatter plots comparing inter-group abundance of key differential gut microbiota at the genus level. **(A)** *Akkermansia*; **(B)** *Romboutsia*; **(C)** [*Eubacterium*]_coprostanoligenes_group; **(D)** [*Ruminococcus*]_gnavus_group; **(E)** *Veillonella*. Normality was tested using the Shapiro–Wilk test, and inter-group differences were analyzed using the Mann–Whitney U test. *p < 0.05; **p < 0.01; ***p < 0.001.

To identify the key microbial taxa driving structural differences between groups, we analyzed community composition and differential abundance at the phylum and genus levels, supplemented by LEfSe analysis for biomarker screening. Analysis of intestinal microbiota community structure and differential species revealed that at the phylum level, the dominant phyla in both groups were Firmicutes, Proteobacteria, Actinobacteria, and Bacteroidetes, collectively accounting for over 96% of the relative abundance. Specifically, the relative abundances of Firmicutes and Bacteroidetes exhibited a slight increase in the treatment group, whereas those of Proteobacteria and Actinobacteria showed a slight decrease; however, these differences were not statistically significant (FDR > 0.05). In addition, six phyla showed significant intergroup differences (FDR < 0.05), among which Tenericutes, Verrucomicrobia, and Cyanobacteria were significantly enriched in the treatment group (Figure 4A). At the genus level, intergroup difference analysis revealed that the abundances of *Akkermansia*, *Romboutsia*, and [*Eubacterium*]_coprostanoligenes_group were significantly increased in the treatment group, while those of [*Ruminococcus*]_gnavus_group and *Veillonella* were significantly decreased (*FDR* < 0.05; Figure 4B). The abundance differences of these genera were further visualized using scatter plots (Figures 5A–E). LEfSe analysis (LDA score > 2.0, *p* < 0.05) identified numerous microbial taxa with significant abundance differences between the two groups. The treatment group was significantly enriched in taxa such as Verrucomicrobiae, Verrucomicrobiales, Akkermaniaceae, and *Akkermansia*, while the control group was significantly enriched in taxa including Veillonellaceae, *Veillonella*, and *Oribacterium* (Figure 4C). These results confirm that the dietary fiber intervention was associated with selective regulation of the abundances of intestinal bacteria, suggesting a modulation of the overall microbiota community structure.

### Predicted relative abundance of significantly altered KEGG level 3 pathways using PICRUSt1

**Figure 6:**
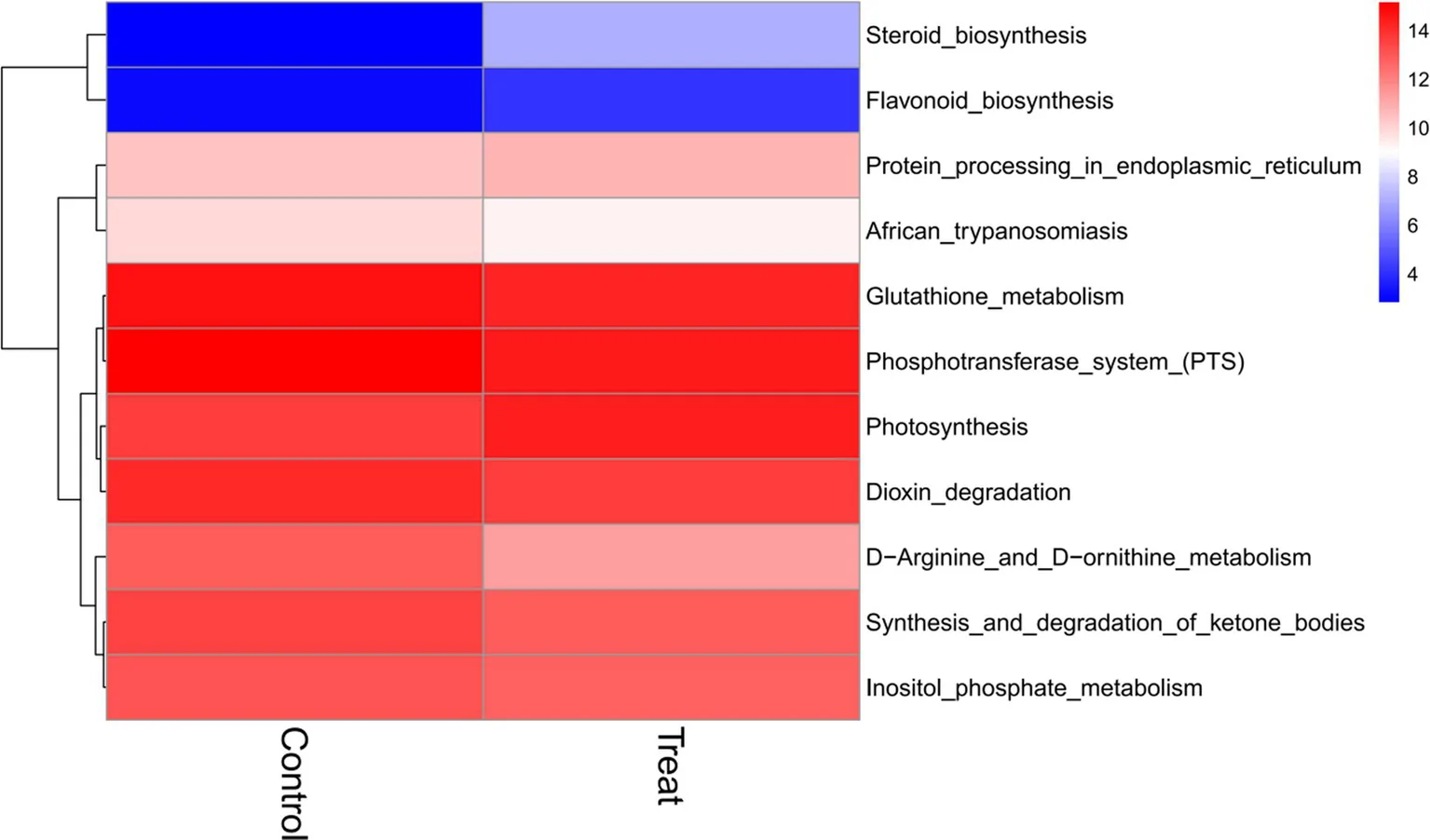
Predicted relative abundance of significantly altered KEGG Level 3 pathways using PICRUSt1. Rows represent predicted functional pathways, and columns represent the control and treatment groups. Color intensity indicates the normalized relative abundance of each predicted pathway (blue: low abundance, red: high abundance). Clustering dendrograms are shown on the left to indicate the similarity pattern of pathway profiles.

Microbial functional potential was predicted using PICRUSt1 at the KEGG Level 3. A total of 11 pathways showed significant differences between the control and treatment groups (*p* < 0.05), which were visualized in a heatmap (Figure 6). The predicted relative abundances of the steroid biosynthesis and flavonoid biosynthesis pathways were markedly higher in the treatment group, with fold changes of 18.41 and 2.02, respectively. Photosynthesis and protein processing in endoplasmic reticulum also exhibited increased abundance in the treatment group. By contrast, most other pathways, including dioxin degradation, phosphotransferase system, glutathione metabolism, inositol phosphate metabolism, ketone body metabolism, amino acid metabolism, and African trypanosomiasis, were significantly downregulated. The clustering dendrogram indicated that these differential pathways clustered into two main groups, reflecting distinct functional profiles between the two study groups.

## Discussion

FD is a common gut-brain interaction disorder without organic lesions, whose pathogenesis is jointly driven by gut microbiota dysbiosis, visceral hypersensitivity and disrupted gut-brain axis communication (Alatorre-Cruz et al., 2026; Sujan et al., 2026; Houghton et al., 2026; Lambiase et al., 2025). Conventional drug therapies have limited efficacy and adverse reactions, while composite fiber represents a safe, non-pharmacological intervention candidate (Gomez-Gomez et al., 2026; Myhill et al., 2026). This double-blind pilot trial systematically integrates *α*-diversity, *β*-diversity, taxonomic composition and functional prediction data to interpret the regulatory effects of standardized composite fiber on intestinal flora and digestive symptoms in adolescents with FD.

The treatment group showed a numerically higher symptom improvement rate, but the intergroup difference did not reach statistical significance (*p* = 0.2246). This discrepancy likely stems from the limited sample size of the pilot study, resulting in insufficient statistical power to detect subtle clinical differences. However, non-significant *p*-values should not be interpreted as definitive evidence against mild clinical benefits of fiber supplementation. α-diversity analysis demonstrated that all four microbial diversity indices were significantly elevated in the fiber intervention group (FDR < 0.05), which suggests composite fiber is associated with a reversal of the low-diversity dysbiotic phenotype typical of FD patients (Zhou et al., 2025). While richer microbial communities are theoretically considered to possess stronger anti-interference capacity and more complete metabolic functions, these observed diversity changes cannot be directly linked to the numerical symptom improvement observed, given the non-significant between-group difference and the lack of formal mediation analysis.

Three complementary *β*-diversity metrics were adopted to comprehensively evaluate shifts in gut community structure and avoid one-sided conclusions drawn from a single analytical algorithm. Weighted UniFrac, which integrates both phylogenetic relationships and species relative abundance, detected obvious intergroup separation of microbial communities (*F* = 68.3, R^2^ = 0.237, *p* = 0.018), while Bray–Curtis dissimilarity only displayed a marginal separation trend and unweighted UniFrac showed no significant group difference at all. This consistent analytical pattern suggests that the intervention primarily influences intestinal flora by altering the relative abundance of existing commensal taxa rather than triggering large-scale gain or loss of microbial species. Stratified PERMANOVA further confirmed that demographic confounders including age, sex, BMI and education explained negligible non-significant variation in community composition across all three distance matrices, and PERMDISP verified homogeneous within-group dispersion between the two arms, thereby supporting the robustness of *β*-diversity statistical outcomes.

At the genus level, fiber intervention significantly enriched *Akkermansia*, *Romboutsia*, and [*Eubacterium*]_coprostanoligenes_group while reducing [*Ruminococcus*]_gnavus_ group and *Veillonella*. Existing cross-sectional case–control studies reported elevated baseline *Akkermansia* and [*Ruminococcus*]_gnavus_group while reduced *Veillonella* in untreated FD patients (Zhou et al., 2025; Wang et al., 2026). Our findings seem to diverge from these baseline observations, as *Akkermansia* showed an increase and *Veillonella* exhibited a further reduction in our treatment group. Notably, multiple pharmacological and dietary interventions that improve FD symptoms have been reported to positively modulate *Akkermansia* abundance (Wang et al., 2026; Chen et al., 2024). This context-dependent dynamic underscores the heterogeneity of gut microbiota alterations in FD and highlights the importance of interpreting microbial changes in the context of intervention rather than cross-sectional comparisons alone. Furthermore, our design cannot confirm whether these shifts are causally linked to the non-significant symptomatic trend. [*Ruminococcus*]*_gnavus_group* has been consistently associated with adverse intestinal microecology and FD-related digestive discomfort (Zhou et al., 2025); thus, its reduction suggests a favorable shift in microbial structure. Regarding *Romboutsia* and [*Eubacterium*]*_coprostanoligenes_group*, current evidence is insufficient to establish their direct roles in FD pathogenesis. These observations underscore the heterogeneous and dynamic nature of gut microbiota alterations in FD and highlight the importance of interpreting microbial changes in the context of intervention rather than cross-sectional comparisons alone.

Functional potential was predicted via PICRUSt1 based on 16S amplicon data, with predicted steroid biosynthesis and flavonoid biosynthesis pathways showing higher relative abundance in the fiber group. This predicted change may correlate with the enrichment of *Akkermansia* and [*Eubacterium*]_coprostanoligenes_group, although such correlations remain speculative without metabolomic validation. *Akkermansia* maintains intestinal barrier integrity and regulates host cholesterol homeostasis, and [*Eubacterium*]_coprostanoligenes_group specializes in converting cholesterol into coprostanol; flavonoids and *Akkermansia* also form a bidirectional regulatory feedback loop, jointly driving the activation of related metabolic pathways (Davey et al., 2023; Mukherjee et al., 2020; Tang et al., 2024; Liu et al., 2025; Watanabe et al., 2023). Nevertheless, all functional inference results are purely computational theoretical outputs without supporting fecal metabolomic detection data to confirm actual intestinal metabolite concentrations, so definitive physiological mechanisms connecting these predicted functional shifts to FD pathology cannot be established. Several predicted pathways also lack clear biological relevance to human intestinal anaerobes, which is an inherent limitation of 16S-based PICRUSt1 functional prediction.

This study has several inherent limitations that should be acknowledged. First, the sample size only meets the standard range of microbiota pilot trials recommended by Whitehead et al. (2016). No formal *a priori* power analysis was conducted in line with pilot study design principles, and the small cohort lacks sufficient statistical power to detect mild clinical differences, requiring larger multi-center trials to verify therapeutic effects. Second, all microbiota data are relative abundance results without absolute quantification via qPCR or flow cytometry, restricting accurate assessment of true microbial load changes. Third, functional prediction solely relied on PICRUSt1 based on 16S sequencing without fecal metabolomic verification, weakening the reliability of mechanistic inference. Fourth, standardized mediation effect analysis was not performed, so we cannot confirm whether gut microbiota changes statistically mediate the relief of digestive symptoms. Fifth, symptom assessment lacked validated Rome IV questionnaires and objective gastrointestinal motility indicators, reducing evaluation accuracy. Sixth, only 28 days of short-term intervention was observed, and long-term follow-up is required to assess persistent microbial regulatory effects. Seventh, the 97% OTU clustering pipeline instead of high-resolution ASV analysis may lower taxonomic identification precision. Eighth, differential taxa screened by LEfSe are merely exploratory without independent cohort validation. Finally, daily diet was only tracked by weekly telephone interviews without standardized food diaries, potentially bringing unmeasured confounding factors to flora composition. The 15 g/d Licun® composite fiber used in this research offers a safe preliminary nutritional intervention; future studies may optimize fiber dosage and formulation, and screen gut microbial biomarkers to achieve individualized dietary guidance for adolescents with FD.

Despite the above methodological limitations, this work fills a prominent literature gap regarding standardized composite fiber intervention targeting gut microbiota in adolescent FD populations. As one of the few double-blind pilot RCTs combining 16S high-throughput sequencing and clinical symptom evaluation for this specific pediatric group, it provides preliminary observational evidence that composite fiber triggers confounder-independent comprehensive remodeling of intestinal microbial ecosystems, accompanied by a numerical trend of relieved digestive discomfort. All clinical inferences drawn from this exploratory research remain tentative and need verification in adequately powered large cohorts. The study delivers feasible nutritional intervention ideas for clinical pediatric digestive health management, and future multi-omics trials integrating absolute microbial quantification and fecal metabolomics are required to resolve all existing limitations and formulate targeted personalized fiber intervention strategies for adolescents with functional dyspepsia.

## Conclusion

In summary, this double-blind placebo-controlled exploratory pilot trial demonstrated that a 28-day standardized composite fiber intervention successfully comprehensively remodeled the gut microbial ecosystem of adolescents with functional dyspepsia independent of demographic confounders. Fiber supplementation was associated with significantly elevated multiple *α*-diversity indices, a reshaped overall community structure, altered relative abundance of several core genera, and higher predicted relative abundance of steroid and flavonoid biosynthesis pathways. These microbial changes occurred alongside a non-statistically significant numerical trend toward alleviating digestive discomfort. Importantly, relevant FD microbiology studies present heterogeneous, context-dependent microbial shifts, so the taxonomic changes detected here cannot be simplistically interpreted as consistent beneficial signatures, and direct causal links between individual bacterial taxa and symptom relief cannot be deduced, especially in the absence of formal mediation analysis. This work provides solely preliminary gut microbiome observational evidence supporting the flora-modulating capacity of composite fiber as a non-pharmacological candidate. Given multiple inherent limitations including small sample size, lack of mediation effect analysis and the absence of multi-omics validation, large-sample multi-center trials integrating absolute microbial quantification, fecal metabolomics and standardized clinical assessment tools are urgently needed to verify therapeutic effects and clarify complex associations between gut microbiota and dyspeptic symptoms.

## Data Availability

The raw 16S rRNA gene sequencing data are available in the NCBI SRA database (https://www.ncbi.nlm.nih.gov/sra) under accession number PRJNA1446699.
